# Single-use solutions for PUPSIT: requirements, challenges, and solutions

**DOI:** 10.1007/s00253-026-13847-5

**Published:** 2026-05-01

**Authors:** Martin Glenz, Peter Eiermann, Britta Manser, Heidi Teschner

**Affiliations:** 1 Cytiva, Dreieich, Germany; 2 Cytiva, Fribourg, Switzerland

**Keywords:** Integrity test, PUPSIT, Single-use system, Sterile filtration

## Abstract

Pre-use post-sterilization integrity testing (PUPSIT) has emerged as a critical standard in biopharmaceutical manufacturing, driven by the revised EU GMP Annex 1 and global regulatory harmonization. PUPSIT aims to verify the integrity of sterilized filters before use, reducing the risks of defect masking and safeguarding drugs against contamination. However, implementing PUPSIT introduces significant technical and operational challenges, including more complex wetting and venting procedures that may bring increased risk of contamination and susceptibility to human error. This review outlines regulatory requirements and risk-based rationales for the PUPSIT evaluation process and discusses the integration of PUPSIT within a broader contamination control strategy. Design strategies for single-use systems emphasize simplicity, error-proofing, and effective wetting and flushing methods. A comparative analysis of single-use PUPSIT systems including manual, shadowboard-assisted, and automated PUPSIT systems illustrates how process flexibility, consistency, and safety can be achieved. Ultimately, successful PUPSIT implementation requires a balance of compliance, operational efficiency, and environmental stewardship, supported by documented risk assessments and validated process controls.

## Introduction to PUPSIT

Pre-use post-sterilization integrity testing (PUPSIT) of critical sterile filters has become the new standard in biopharma drug manufacture, reflecting a significant evolution in sterile filtration assurance. Since the revised “EU GMP Annex 1: Manufacture of Sterile Medicinal Product” on filter integrity testing in 2022, drug manufacturers are expected to determine filter integrity not just after filtration, but also before filtration, after the filter has been sterilized. The driving force for performing PUPSIT is to safeguard patient safety: to detect the presence of a non-integral filter after sterilization, before the process fluid, or impurities in the process fluid, could potentially mask a filter damage or defect. Such masking would increase the risk of the marginal damage not being detected by the post-use integrity test, which carries a safety risk for patients.

Implementing PUPSIT is not without considerable technical and operational challenges, as a survey of drug manufacturers by BioPhorum Operations Group in 2024 shows: an integrity test before use requires a wetting and venting of the filter, followed by pressurized air being subjected onto the filter for the integrity test measurement, typically either bubble point or forward flow testing. The additional connections for these operations can bring extra risks of dead legs, hold-up volume, or non-conformities, which present a risk especially on the sterile downstream side of a filter. Industry feedback highlighted concerns with these complexities including system design, reliable filter wetting, and the increased risk of human error in the more intricate PUPSIT process (BioPhorum Operations Group (BPOG), 2024).

To safely implement PUPSIT into the final sterile filtration step of drug product manufacture, special design considerations are to be taken. This includes taking the right choice of components, design strategies for safe installation, flushing, venting, and testing, as well as automation approaches to ensure process consistency and robustness. This review article highlights best practices and key aspects for successfully implementing PUPSIT such as material and component selection, flow kit design, and strategies for handling and process control.

## Regulatory requirements for PUPSIT

The EU GMP Annex 1 introduced explicit requirements for PUPSIT. Section 8.87 states: “The integrity of the sterilized filter assembly should be verified by integrity testing before use (pre-use post sterilization integrity test or PUPSIT), to check for damage and loss of integrity caused by the filter preparation prior to use.” This requirement addresses the risk of filters being damaged during transport, unpacking, installation, or sterilization as well as filter flaw masking, where defects could be obscured during filtration and escape detection in post-use tests. It also mandates that filter integrity tests be validated and correlated with microbial retention capability. Common methods include bubble point, diffusive flow, water intrusion, and pressure hold tests (European Medicines Agency (EMA), 2022). This new clarity in the regulatory intent to both reinforce the guidance and to ensure that risk assessments, and the good science that informs them, underpins the chosen action. The new revision has also been adopted by PIC/S (Pharmaceutical Inspection Co-operation Scheme), which now also requires drug manufacturers outside of the EU to assess and implement PUPSIT. While PUPSIT may not be strictly mandatory, drug manufacturers are expected to assess and explain any exceptions very carefully in a risk assessment.

In the context of a broader contamination control strategy (CCS), where risks are assessed and managed according to quality risk management (QRM) principles described in ICH Q9 (European Medicines Agency (EMA), 2025), PUPSIT represents a mitigation strategy. QRM ensures that decisions regarding filter integrity testing are science-based, systematic, and documented, reducing the risk of microbial contamination in sterile medicinal products. Annex 1 emphasizes that manufacturers must apply QRM principles, as outlined in ICH Q9, to justify their approach to PUPSIT. This involves identifying risks associated with filter integrity, assessing their impact on sterility assurance, and implementing proportionate controls. The framework requires documented evidence that sterilization processes, handling, and assembly do not compromise filter integrity. Therefore, risk assessments for PUPSIT should consider factors throughout the life cycle of a filter, starting with filter design and manufacture, the transportation, and storage of a filter to the point it is unpacked and installed at the point of use. It should also consider possible risks that occur from the sterilization method (e.g., steam-in-place, autoclaving), filter flushing and the filtration process itself, as well as product-specific characteristics, and overall process complexity. Table 1 gives a brief overview of possible risks that should be assessed. Examples for risk assessments and more detailed considerations to PUPSIT have been published by Joseph (2023), Waldron et al. (2025), Salamatian et al. (2025) and Pierno (2024).

**Table 1.**
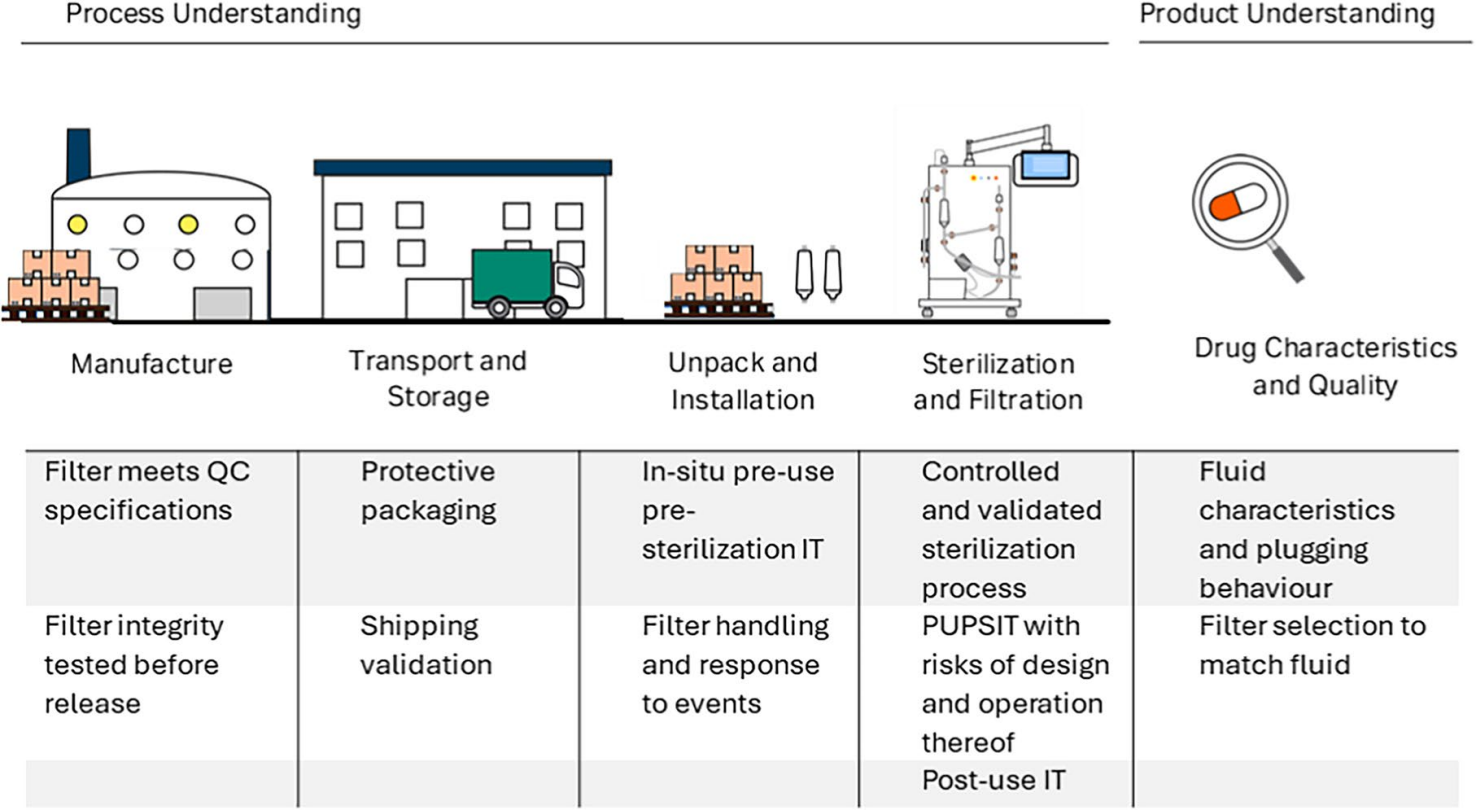
Considerations for a risk assessment for sterile filtration as part of contamination control strategy

Annex 1 acknowledges that PUPSIT may not always be possible. Processing of very small volumes is given as example, but volumes are not specified. In such cases, a thorough risk-based rationale must be documented, supported by process knowledge and supplier data. Important factors when performing a process risk assessment are the chance of a present filter damage being masked and whether the process fluid can plug and thereby mask this damage (Ferrante et al. 2020). Regulators expect clear evidence that the chosen approach maintains sterility assurance and complies with GMP principles.

From a global perspective, regulations for aseptic processing slightly differ. In the USA, the Food and Drug Administration’s (FDA) guidance (2004) requires post-use integrity testing for batch release but does not mandate PUPSIT and does recommend it as a best practice however. The approach is risk-based under 21 CFR 211.113(b), allowing flexibility; the provided sterility assurance is maintained. Other major regulatory bodies align with Pharmaceutical Inspection Co-operation Scheme (PIC/S) principles (2023), which includes Health Canada (2025), the Japanese Pharmaceuticals and Medical Devices Agency (2011), the South Korean Ministry of Food and Drug Safety (MFDS 2016), and the Chinese National Medical Products Administration (NMPA 2011). Even when not naming PUPSIT, these regulatory bodies expect integrity testing of sterilizing-grade filters pre- and post-use and clear documentation as a vital part of the contamination control strategy. This indicates a global trend towards harmonization in aseptic processing.

## Single-use systems for PUPSIT

Single-use technology (SUT) and stainless-steel filtration systems are two commonly used options in biomanufacturing. While processes were historically built in steel facilities, SUTs are now widely used across the entire product life cycle from development to commercial manufacture. Single-use flow kits for PUPSIT with sterile filters are supplied gamma irradiated and thus ready for flushing, integrity testing and filtration. Different methods for integrity testing are available to the industry as described in the “Mechanical strength and assembly integrity” section of this review; however, regardless of the test method, the wetted filter is pressurized with air, and the diffusive gas flow through the membrane is measured. In addition to verifying the filter integrity, selected integrity test devices also support pressure-hold testing of the entire single-use flow kit and flush bags to verify system tightness prior to processing (Bio-Process Systems Alliance (BPSA), 2023).

### Design strategies for single-use PUPSIT systems

PUPSIT should reduce patient risk, not increase it; therefore, a key element in final filtration is having a reliable and robust single-use system (European Commission 2022). Simplicity in design is essential, keeping the system intuitive, and error-proof is critical to ensure consistent performance (BioPhorum Operations Group (BPOG) 2021). The flow kit should follow Poka-Yoke principles, which are mistake-proofing techniques that ensure a process or product is designed in such a way that human error is either impossible or immediately detectable. This prevents assembly errors both at the supplier and during installation or operation at the drug product manufacturing site. The design must go beyond just the filtration process; it should also support effective filter wetting and flushing. The flush liquid can either be collected in a biocontainer or exit the filter flow kit by using hydrophobic/hydrophilic barrier filters.

**Fig. 1:**
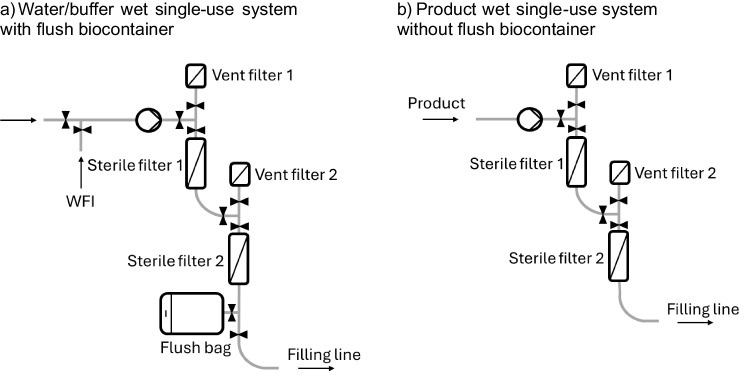
Examples for single-use systems with **a** water/buffer wetting and** b** product wetting

**Fig. 2:**
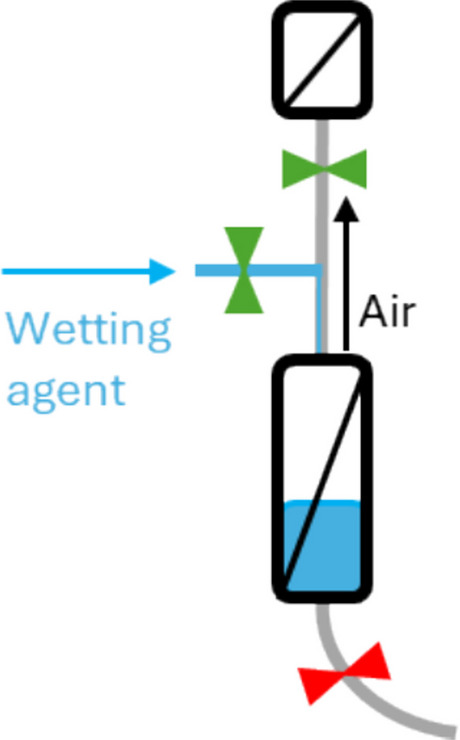
Schematic drawing of vertical venting

The design of a PUPSIT flow kit is heavily influenced by the wetting agent used to wet the filter membrane prior to integrity testing. For wetting with water, an additional water inlet must be integrated as well as a bigger flush bag to collect the water after wetting (Fig. 1a). For product-wet processes, the system design can be held simpler as only one product inlet is required, and a flush bag becomes optional (Fig. 1b). In both scenarios, air must be removed from the capsule during wetting and priming before and after the integrity test. In the first wetting step of PUPSIT where the membrane is still dry, the air can easily pass through the dry sterile filter membrane and leave the flow kit downstream of the filter. However, after complete wetting of the membrane and pre-use filter integrity test, the air can be only removed from the filter capsule via (1) the manual vent valves directly on the capsule or (2) vertical venting using the vent filter connected upstream of the liquid filter (Fig. 2). Option 1 requires, for example, an attached flush bag on the vent valves of the filter to collect air and droplets of the fluid; otherwise, there is a risk of contamination of the system and a risk of leaking the product into the cleanroom or isolator. Venting through dedicated vent filters is a safe and convenient option since the air filters are also needed to connect the integrity test machine when performing the filter integrity test. The orientation of both sterile and vent filters is important to maintain vertical venting of the system.

Design principles aim to eliminate areas where fluids can stagnate, such as dead legs, which do not easily allow for full product recoveries. Components should be engineered to support complete drainage, including sloped surfaces, radial flow paths, and the use of aseptic connections that reduce the risk of contamination during assembly. Weld-free, pre-assembled single-use systems therefore offer additional benefits by simplifying installation and reducing the potential for human error.

In small batch manufacturing, maximizing product recovery is essential. However, achieving both high recovery and a reliable wetting procedure can be challenging to integrate effectively in one single-use system. The choice of membrane type and filter size, tubing diameter, and recovery methods impacts the product recovery. For example, introducing sterile air into the capsule’s inner core to facilitate recovery is a complex task. Incorporating a sterile air filter downstream of the primary sterile filter adds a critical component that requires post-use validation and could risk a batch. Bory et al. (2015) discussed strategies and best practices to wetting for integrity testing in volume-restricted processes.

### Material requirements for single-use PUPSIT systems

The materials used in PUPSIT assemblies should be thoughtfully chosen and qualified to withstand sterilization processes and maintain their functional integrity throughout the product life cycle as shown in Table 1. As part of this life cycle, materials must be resilient to the desired sterilization methods, high pressure and chemical exposure of the drug and excipients. It is essential that these materials retain their functional properties to ensure the sterility and reliability of the process. This includes maintaining mechanical strength, dimensional stability, and a low amount of extractables and leachables that could contaminate the product. With increasing focus on environmental footprint of drug manufacturing, sustainable materials and designs for single-use systems gain importance.

Compliance with international standards and industry guidelines is key to aligning materials and design with a robust CCS. Additional standards such as ISO 13408-1 for aseptic processing (International Organization for Standardization (ISO), 2023), ISO 11137 for radiation sterilization (International Organization for Standardization (ISO), 2025), and USP chapters on microbial and particulate contamination provide a solid framework for ensuring product safety and efficacy (United States Pharmacopeial Convention (USP), 2025). Industry-specific recommendations, such as those from DECHEMA (DECHEMA Expert Group Single-Use Technologies 2020) and validation guides from suppliers, offer practical insights into the selection and qualification of materials for single-use systems.

In pharmaceutical manufacturing, especially within the framework of PUPSIT, the scrutiny of materials for extractables and leachables (E&L) is not just a regulatory checkbox; it is a matter of patient safety and product integrity. These chemical entities, which can migrate from materials into the drug product, must be thoroughly understood and well controlled. Extractables are compounds that can be drawn out of a material under exaggerated conditions, such as elevated temperature or aggressive solvents. Leachables, on the other hand, are those compounds that migrate into the drug product under normal processing or storage conditions. The distinction is subtle but critical: extractables help predict what might leach, while leachables are what actually do.

The evaluation of E&L typically begins with a well-designed extraction study, and this involves exposing the material to solvents under controlled conditions and analyzing the resulting solutions using techniques like gas chromatography-mass spectrometry (GC-MS), liquid chromatography-mass spectrometry (LC-MS), and inductively coupled plasma mass spectrometry (ICP-MS). These methods are very sensitive and allow scientists to detect even trace levels of organic and inorganic compounds.

Regulatory bodies such as the FDA and European Medicines Agency (EMA) expect manufacturers to follow established guidelines, including USP < 665 > (United States Pharmacopeial Convention (USP), 2022), ISO 10993 (International Organization for Standardization (ISO), 2023), and the BioPhorum Operations Group (BPOG) best practices. These frameworks provide a structured approach to assessing the safety of materials used in contact with drug products. Regulatory standards such as ISO 10993−1 (latest revision) (International Organization for Standardization (ISO), 2025) (International Organization for Standardization (ISO), 2023), and BPOG protocols (BioPhorum Operations Group (BPOG), 2022) (BioPhorum Operations Group (BPOG), 2020) (BioPhorum Operations Group (BPOG), 2025) offer guidance for evaluating extractables and leachables, ensuring that materials meet the stringent requirements of pharmaceutical manufacturing.

However, a robust E&L strategy is not just about testing; it is about integrating material science with process design. It involves selecting the right materials, understanding their behavior under sterilization and operational conditions, and ensuring that any potential leachables are within safe limits. This strategy must be embedded in the design, qualification, and validation of single-use systems. Ultimately, the goal is to ensure that nothing from the material compromises the drug product. In a world where patients rely on the purity and safety of every dose, the vigilance around E&L is an important guardian of quality. Suppliers of SUT also offer product-specific generation of E&L profiles as a service, which can be used by the end users in a risk-based approach to determine the level of these substances per dose of final product.

#### Mechanical strength and assembly integrity

The mechanical integrity of PUPSIT assemblies is essential for ensuring operational reliability and maintaining contamination control. These systems must endure the physical stresses imposed during sterilization, integrity testing, and routine handling, all while preserving a sterile barrier.

For system sterilization such as gamma or X-ray irradiation, materials are exposed to significant chemical stress due to the breaking of covalent bonds in polymers, in other words, the creation of free radicals. This in turn leads to chain scission, crosslinking, and uneven structural changes that generate internal stress. Therefore, these processes can alter the physical and chemical properties of polymers, potentially compromising their integrity and performance. Materials like polyethersulfone (PES), polyethylene (high-performance polyethylene (HPPE), ultra-high molecular weight polyethylene (UHMWPE)), and polyvinylidene fluoride (PVDF) are commonly used due to their exceptional chemical resistance, thermal stability, and low permeability. These materials retain their mechanical strength and microbial barrier function even after gamma irradiation, making them ideal for sterile filtration systems. Other materials such as polyether ether ketone (PEEK), polypropylene (PP), and silicone also play important roles, with PEEK providing excellent mechanical and thermal properties for high-performance connectors and housings, and silicone offering flexibility and biocompatibility, though it requires thorough evaluation for extractables (Kushwah, et al. 2022; Huang, et al. 2011; Jenke et al. 2006).

During integrity testing, PUPSIT assemblies are potentially exposed to pressures that exceed 4 bar (e.g., for bubble point integrity tests). To withstand these conditions, tubing materials must offer both flexibility and high tensile strength, so reinforced silicone and braided thermoplastic elastomers (TPE) are commonly used because they maintain structural integrity under pressure and are compatible with most sterilization methods. However, connections and junctions within the assembly are particularly vulnerable to mechanical failure or leakage. To mitigate these risks, secure clamping mechanisms such as Oetiker™^1^ clamps are employed to ensure tight seals while overmolding techniques further enhance reliability by eliminating crevices and potential leak paths, thereby improving both mechanical strength and hygienic design (Bio-Process Systems Alliance (BPSA), 2023).

Mechanical robustness is validated through standardized testing protocols. Burst pressure testing determines the maximum pressure the system can withstand before failure, while tensile strength testing evaluates the material’s resistance to pulling forces. These tests follow international standards such as ASTM D412 (ASTM International 2021) and ISO 37:^2024^ (International Organization for Standardization (ISO), 2024), which provide guidelines for assessing the performance of elastomeric and thermoplastic materials.

Mechanical strength is not an isolated requirement, but a vital component of the broader CCS. Assemblies that fail under pressure or mechanical stress can compromise sterility, introduce contaminants, and disrupt process continuity. Therefore, mechanical integrity must be considered during the design, material selection, and validation phases of PUPSIT system development.

#### Integrity testing (IT)

Integrity testing of single-use assemblies broadly falls into two categories: general integrity tests, which use pressure-decay, vacuum-decay, or gas-flow methods to identify gross and moderate leaks in a practical, non-destructive manner; and helium-based integrity tests, which provide high-sensitivity detection of micro-defects and establish a direct, quantitative link between physical leakage and microbiological risk.

##### General integrity tests

Maintaining the integrity of single‑use assemblies is essential for reliable and sterile bioprocessing. One of the most practical options for IT is pressure‑decay testing, in which the assembly is pressurized and observed for any loss of pressure over time; semi‑automated systems have improved both sensitivity and consistency, making this method widely applicable in routine operations (Waldron et al. 2025).

A related approach, vacuum‑decay testing, evaluates changes in vacuum rather than pressure and is recognized among standard leak‑testing tools, particularly for components that tolerate vacuum without deformation (ASTM International 2024). Mass‑flow leak testing offers another useful pathway and measures the gas required to maintain stable pressure conditions. It is widely used for applications involving larger leak rates. Vendor and engineering‑method documentation show that mass‑flow approaches are particularly suited for higher‑flow or large‑volume components, while high‑range mass‑flow methods are specifically applied when large leaks are present and fine‑defect sensitivity is less critical (Hogreve 2023).

Non‑tracer approaches using air or nitrogen pressure/flow methods provide economical, non‑destructive options where moderate sensitivity suffices. ASTM E3336‑22 (ASTM International 2023) explicitly standardizes physical integrity testing of single‑use systems and details pressure‑based methods and apparatus/validation requirements for empty, dry SUS, enabling routine integrity/leak testing without specialty tracers. This aligns with the PDA body of work that correlates gas‑flow behavior through micro‑defects with maximum allowable leakage limits (MALL) (Hogreve 2023) and demonstrates that validated gas‑flow measurements can be mapped to microbiological risk thresholds, thereby justifying air/nitrogen tests in use‑cases that do not demand ultra‑trace sensitivity.

Complementing these physical methods, careful visual inspection of welds and junctions remains a cornerstone of quality assurance. Finally, ASTM E3244‑23 encourages a risk‑based selection of these methods to match the criticality of each SU application (ASTM International 2023).

##### Helium integrity test

The helium integrity test is the most widely validated, high‑sensitivity, lifecycle‑aligned method that directly links a measured physical signal to microbiological risk; it deserves explicit treatment separate from general pressure/flow tests (Bio-Process Systems Alliance (BPSA), 2019). Helium integrity testing is a highly sensitive and non-destructive method for detecting microleaks in single-use systems (United States Pharmacopeial Convention (USP), 2025). Its application is especially valuable for single-use bags and flexible containers, where traditional testing methods may lack the sensitivity required to identify minute breaches.

The test method and the principle behind it use the properties of helium gas: due to its small molecular size and inert nature, helium can penetrate even tiny imperfections in a material or weld seam. This makes it an ideal tracer for leak detection, capable of identifying breaches that would otherwise go unnoticed using conventional pressure decay or bubble emission techniques. The method and acceptance criteria have been fully validated by some suppliers to detect a defect down to a 2 µm with assemblies including biocontainers ranging in size from 50 mL to 200 L and mixer biocontainers in sizes up to 200 L (Bio-Process Systems Alliance (BPSA), 2023). For filling set assemblies, the method is validated to detect defects down to 10 µm. Both comply to the MALL (maximum allowable leakage limit, defined in USP 1207 (United States Pharmacopeial Convention (USP), 2025) (ASTM International 2023). The implementation of helium leak testing is guided by standards such as ASTM E3244-23, which outlines procedures for calibration, sensitivity verification, and validation of helium leak detection systems, and these protocols ensure consistency and reliability across different testing platforms and applications.

However, the high sensitivity of helium testing also imposes stringent requirements on the materials used in single-use assemblies. Films and components must exhibit extremely low helium permeability to prevent false failed and ensure accurate results, so high-barrier multilayer films, often incorporating ethylene vinyl alcohol (EVOH) and polyvinyl chloride (PVC)–polyvinylidene chloride (PVDC) layers to help achieve good gas barrier properties, are commonly employed to meet these demands (López-Rubio 2011).

Automated helium integrity testing platforms are increasingly being adopted in manufacturing environments at suppliers; these systems reduce operator variability and improve detection accuracy. By integrating helium leak testing into the overall CCS, manufacturers can significantly reduce the risk of undetected leaks and ensure the robustness of single-use systems used in aseptic processing.

In summary, helium integrity testing represents a powerful tool in the validation and quality assurance of PUPSIT assemblies. Its ability to detect microleaks with high precision supports the broader goals of sterility assurance and contamination control in pharmaceutical manufacturing.

#### Sustainability and environmental considerations

Sustainability is becoming a key design criterion for single-use systems. Manufacturers are increasingly adopting recyclable materials and modular designs to reduce waste (Shann 2024) according to the 17 goals established by the United Nations (UN) and adopted by all UN member states in 2015 (United Nations (UN), 2015). Scalable bag systems that minimize wetting fluid waste and support energy recovery through incineration are gaining traction. Bio-based thermoplastic elastomers (TPEs) and recyclable polyethylene films that meet aseptic processing standards while reducing environmental impact have been developed. These materials are evaluated using ISO 14040:^2006^ and ISO 14044:^2006^ (International Organization for Standardization (ISO), 2006) for life cycle assessment and for recyclability.

Next-generation sterilizing-grade filters are increasingly subject to sustainability evaluations aligned with internationally recognized standards, such as ISO 14067 for carbon footprint quantification (International Organization for Standardization (ISO), 2018), enabling end users to systematically assess and optimize the environmental impact of their filtration processes.

On another front, environmental regulatory pressure in multiple jurisdictions is accelerating the transition away from PFAS (per‑ and polyfluoroalkyl substances) materials (ECHA European Chemicals Agency 2023). Currently, all biopharmaceutical products developed or licensed in the USA utilize PFAS materials in one or multiple steps of the development or production process (BioPhorum Operations Group (BPOG), 2025). While the impact of proposed PFAS regulations to the biopharmaceutical industry is not yet clear, there are currently no approved restrictions directly impacting fluoropolymers in bioprocessing. Any future restrictions, if implemented, are fully expected to allow lengthy timelines for the industry to develop, qualify, and transition. Sector organizations including BPSA and BPOG argue for differentiated regulatory treatment to avoid unintended disruptions to essential single‑use components required for biologics manufacturing.

## Handling, automation, and control strategies

This section contrasts three prevalent approaches to PUPSIT in sterile filtration across handling practices and enabling automation: (1) manual single-use PUPSIT systems, (2) manual shadowboard PUPSIT systems, and (3) fully automated PUPSIT systems. PUPSIT assemblies and systems are available by a range of suppliers (such as Cytiva, Meissner Corporation, Merck KGaA, Parker, Saint-Gobain Biopharma, Sartorius AG, Single Use Support, and others) in various designs, formats, and material of construction.

### Manual single-use PUPSIT systems

In a manual single-use PUPSIT configuration, operators assemble presterilized single-use flow paths, install sterilizing-grade capsules or cartridges, connect a portable integrity tester, wet the membrane, execute the integrity test, and document outcomes. The primary strengths of this approach are flexibility and, in relation to more automated system options, low capital cost, which can be attractive for development or low-throughput operations. However, the approach inherently increases operator dependency for critical manipulations on both upstream and downstream sides of the sterilizing filter during wetting, venting, and test gas evacuation. These are recognized as operations that can elevate contamination risk if not designed and executed well and within a robust CCS.

From a control perspective, manual single-use PUPSIT leans on procedural controls: validated standard operating procedures (SOPs), operator training, checklists, and independent verification to ensure correct wetting, parameter entry, and limit checks. The absence of closed-loop automation means that consistency depends on human performance. EU inspectors increasingly expect these procedural elements to sit inside a documented CCS and QRM framework. These should be consistent with ICH Q9, which necessitates formal risk assessments for choices such as test location, and any justified omission of PUPSIT where volumes are extremely small.

### Manual shadowboard PUPSIT systems

A shadowboard system is still manual but introduces a standardized, visual layout for components, ports, valves, and disposable subassemblies to guide assembly and testing. The benefit is a reduction in assembly variability and setup errors, increased product recovery and product yield, improved ergonomics, and faster, more reproducible execution relative to ad hoc manual single use-rigs (International Society for Pharmaceutical Engineering (ISPE), 2018). In practice, shadowboard embed connection sequences, color-coding-, and fixed mounting points for the integrity tester connection, drain and vent bags, sterile connectors, and pressure gauges. The approach remains subject to the same downstream sterility controls as fully manual rigs, and it still requires high-quality training and supervision. Nevertheless, by constraining a certain degree of assembly freedom, shadowboard are often a practical bridge between fully manual and fully automated stations in GMP settings. Offline or benchtop testing of housings can also be used to minimize production line downtime, a concept where a shadowboard can support by enabling multiple housings to be prepared for testing in parallel (Stering 2014).

Control strategies in shadowboard systems remain predominantly procedural but can incorporate simple interlocks and poka-yoke features embedded in the board design and standardized wetting sequences.

### Fully automated PUPSIT systems

Fully automated PUPSIT systems integrate assembly-specific single-use flow kits, automated priming and wetting phases, pressure ramping under closed loop control, instrumented venting and drainage, and execution with electronic batch records and audit trails driven by recipes. These features minimize manual interventions on the sterile side, improve repeatability of test conditions, and embed alarm handling and interlocks that halt execution or trigger preprogrammed mitigation upon deviation. Suppliers of those machines describe systems that natively execute both PUPSIT and post-use integrity tests and can incorporate leak tests of the single-use flow path prior to product commitment, aligning tightly with Annex 1 while reducing contamination risk from handling. Automation also facilitates real-time parameter verification against validated ranges and direct manufacturing execution system (MES) connectivity for contemporaneous documentation. The net effect is risk redistribution from operator variability and handling towards design qualification and software validation.

### Comparing outcomes across the three approaches

When looking at the intent of Annex 1 and what inspectors focus on, manual single-use systems can meet the requirements, but they lean heavily on operator skill, environmental discipline, and meticulous documentation. They tend to the needs in lower-volume settings or where agility really matters, provided every design choice is backed by a strong CCS and QRM rationale.

Shadowboard setups can be seen as a smart middle ground. They do not automate the process, but by standardizing the layout and physically encoding the process steps, they cut down on assembly errors and make execution more consistent. Additionally, they make conversations with inspectors easier because users can point to a tangible, standardized practice designed to control risk. It is a way to close part of the gap to automation without a big capital spend.

Fully automated systems tick all the boxes for data integrity, repeatability, and closed-system ideals. They minimize contamination risk, speed up setup and testing, and handle electronic records natively. The trade-off is higher upfront investment along with the need for formal software validation and recipe lifecycle management.

Independent from the approach to implementing PUPSIT as shown in Table 2, the inspection questions do not change: Can the control strategy clearly prevent or detect a non-integral filter before product exposure? Do data show validated correlation between test results and microbial retention? And is there a risk-based rationale for every deviation from default expectations? Those are the conversations that matter.

**Table 2.**
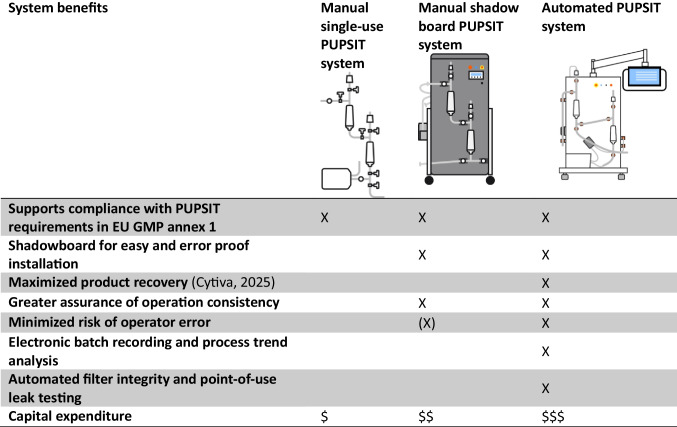
Comparing outcomes across the three approaches to PUPSIT systems

Across the three implementation modalities (manual, shadowboard-standardized, and fully automated), capital expenditures (CAPEX) differ materially when equipment scope, control architecture, and validation requirements are considered. By contrast, operational expenditures (OPEX) are broadly comparable under equivalent throughput and staffing assumptions.

Despite these economic nuances, fully automated systems remain justified, as mentioned, on the basis of application safety, offering superior repeatability, data integrity, and risk control consistent with contemporary expectations for contamination control and closed processing.

## Conclusion

The implementation of PUPSIT in critical sterile filtration has become a critical requirement and marks an advancement in quality assurance within biopharmaceutical manufacturing. By detecting filter damage after sterilization but before use, PUPSIT mitigates the risk of defect masking during filtration and helps prevent product contamination, thereby strengthening sterility assurance. Since decades and even more now with Annex 1 updated in 2022, the biopharmaceutical industry, including drug manufacturers, suppliers, and industry groups, have developed a range of options and best practices for system design, material selection, and process operation to meet these new requirements around performing PUPSIT. Technical challenges can be addressed by best practices in process design and material selection. Single-use system options that allow for either a manual, shadowboard-based or fully automated PUPSIT and filtration process offer simplicity and robustness and enable a successful implementation of a safe and effective PUPSIT strategy.

Looking ahead, the role of PUPSIT is expected to further evolve as advances in single-use technologies, smart assemblies, and integrated automation with enhanced data integrity continue being implemented. Ongoing dialogue between regulators, industry, and suppliers remains essential to clarify and harmonize expectations and enable risk-based and reliable approaches to implementing PUPSIT. As experience with and acceptance of PUPSIT continue to grow across diverse processes and modalities, it is anticipated that standardized, user-friendly solutions will further embed PUPSIT as a routine and value-adding element of sterile manufacturing, supporting both patient safety and operational efficiency.

## Data Availability

Not applicable. This manuscript does not report data generation or analysis.

## Footnotes

1 Oetiker™ is a registered trademark of Oetiker Group.
